# A Rare Finding of Pulmonary Epithelioid Hemangioendothelioma

**DOI:** 10.1155/crip/1830357

**Published:** 2026-10-15

**Authors:** Riona Park, Curtis Ober, Calista Sha, Hailey Lau, Isabella Sha, Alain Borczuk, Paul C. Lee

**Affiliations:** ^1^ Department of Thoracic Surgery Northwell Health Long Island Jewish Medical Center New Hyde Park New York USA; ^2^ Department of Pathology Northwell Health; Donald and Barbara Zucker School of Medicine at Hofstra/Northwell 500 Hofstra University Hempstead New York USA

**Keywords:** case report, epithelioid hemangioendothelioma, lung cancer, pulmonary EHE, wedge resection

## Abstract

Epithelioid hemangioendothelioma (EHE) is a rare vascular tumor with variable presentation and malignancy. These highly uncommon tumors may arise from the liver, soft tissues, bones, and, more infrequently, from the lungs. Given its rarity, treatment approach is not unanimous, with ongoing debate regarding the relative benefits of surgical resection and radiation therapy. In support of growing efforts to standardize treatment of these tumors, we present our findings of a rare pulmonary EHE, incidentally detected in the left lung of a 65‐year‐old, nonsmoking female patient with a history of diabetes, hypertension, and renal cell carcinoma (RCC) in remission for 8 years after surgical resection. A surgical route was recommended, and left robot‐assisted thoracoscopic wedge resections were performed. Final pathological assessment confirmed two pulmonary EHE tumors, one resected from the left upper lobe (LUL) and another from the left lower lobe (LLL) of the lung. Her postoperative course was uneventful, and at 13 months postresection, there has been no evidence of recurrence.

## 1. Introduction

Epithelioid hemangioendothelioma (EHE) is a highly rare vascular sarcoma representing less than 1% of all vascular tumors [1]. With an incidence rate of 0.23 per 1,000,000 person‐years, EHE demonstrates a slight predominance in females and in patients aged 60–79 years [2]. The majority of EHE develops from the skin and soft tissues, followed by the abdomen. EHE presentation and its clinical behavior vary greatly. With minimal prognostic factors, EHE findings are often incidental with metastatic disease observed in over 50% of cases by the time of presentation [3, 4].

EHE of pulmonary origin is estimated to comprise 13% of total EHE incidence [2]. When originating from the pulmonary tissue, these lesions have been colloquially referred to as pulmonary or thoracic EHE. Pulmonary EHE typically manifests multifocally, and radiographic findings appear with three different subtypes: multiple pulmonary nodules, multiple pulmonary reticulonodular opacities, or diffuse infiltrative pleural thickening [5–7]. There is no established treatment of choice for pulmonary EHE, so surgery and/or chemoradiation is considered on a case‐by‐case basis. The behavior of metastatic EHE can vary, ranging from low‐grade malignancy to that of a high‐grade sarcoma. Localized EHE typically behaves as a low‐grade malignancy despite a high propensity for systemic involvement.

Given how little is understood about these tumors, clinical approach has been tentatively constructed from case reports and limited retrospective case series. To contribute to the ongoing need for standardized treatment and management protocols, we present our findings of multifocal pulmonary EHE which was surgically resected from two separate lobes of the lung in a 65‐year‐old nonsmoking female with a previous history of renal cell carcinoma (RCC).

## 2. Case Presentation

A 65‐year‐old nonsmoking female patient presented to our thoracic surgery clinic after multiple new juxtapleural pulmonary nodules were found during surveillance medical imaging. She was asymptomatic, denying respiratory and systemic symptoms such as cough, shortness of breath, fever, and chills. The patient had a history of hypertension and diabetes. The patient notably also had a history of RCC for which she received a radical left nephrectomy 8 years prior. Since then, the patient was under regular observation with urology without evidence of malignant disease throughout her routine abdominal ultrasounds and CT scans over the years. However, although no evidence for local recurrence of RCC was found, her most recent chest CT scan captured new pulmonary nodules in the left lung, including a 6 mm nodule in the posterior left upper lobe (LUL), and another in the left lower lobe (LLL). A subsequent skull‐to‐thigh PET/CT was performed, and the 6 mm nodule in the posterior LUL demonstrated minimal FDG‐avidity (SUV 1.9), whereas the other was either not avid or below the resolution limit of PET. No extrathoracic involvement was observed. The development of new lung nodules prompted suspicion for late metastatic recurrence of RCC. A surgical route was offered, and the patient′s pulmonary function testing demonstrated adequate lung reserves. An elective left robot‐assisted thoracoscopic procedure consisting of wedge resections of the LUL and LLL with mediastinal lymph node dissection was performed without complications.

Two foci of EHE originating from lung parenchyma were ultimately confirmed and supported by immunohistochemical analysis. Histopathological evaluation of the frozen tissue blocks showed well‐circumscribed nodules abutting the pleura. Resected wedge samples were sent to pathology for intraoperative as well as final pathological examination. From the LUL, a firm well‐circumscribed mass (0.8 × 0.7 × 0.6 cm) underlying an area of pleural puckering and abutting the pleura 1.0 cm away from the resection margin was identified. The separate wedge resection from the LLL contained a slightly larger, similarly well‐circumscribed lesion (1.0 × 0.6 × 0.5 cm), which also abutted the pleura and was 0.4 cm from the resection margin (Figure 1A). The lesions were analyzed separately then compared. Upon final pathological analysis, the lesions were seen to share similar growth patterns. Cells were eosinophilic, commonly seen with EHE tumors, and diagnosis was further confirmed upon immunostaining assessment (Figure 1B) [8]. Immunohistochemical analysis confirmed positive expression of vascular markers CAMTA1 (Figure 1C) and ERG (Figure 1D). Both tumor cells were also positive for CD34 and CD31, further supporting endothelial origin. Both were negative for S100, SOX10, INSM1, CAM5.2, CD117, desmin, and smooth muscle actin (SMA). Necrosis was observed in one of the nodules, which contained an area of central necrosis comprising approximately 40% of the nodule (Figure 2A). Mitotic activity was low, as a review of 30 high power fields revealed two mitotic processes, one of which is shown in Figure 2B. All dissected lymph nodes were negative for malignancy.

**Figure 1:**
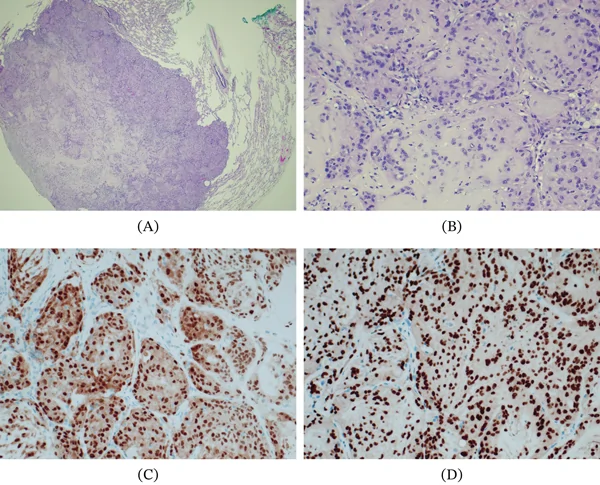
(A,B) Microscopy of the collected specimen from the left lower lobe (1.0 × 0.6 × 0.5 cm) stained with hematoxylin & eosin (H&E). (A) EHE lesion abutting the pleura (H&E 5x). (B) EHE cells with eosinophilic cytoplasm (H&E 100x). (C,D) Immunohistochemical analysis. (C) Positive CAMTA1 expression (150x magnification). (D) Positive ERG expression (150x magnification).

**Figure 2:**
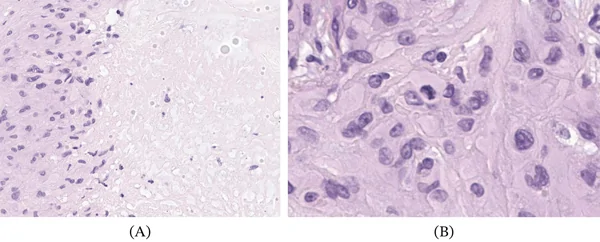
(A,B) Hematoxylin and eosin (H&E) stain of lesion resected from lung. (A) One nodule with region of central necrosis making up approximately 40% of the lesion (H&E 100x). (B) Rare mitotic activity; two mitoses observed upon review of 30 high power fields, one of which is shown (H&E 50x).

The patient′s postoperative course was uneventful. Her follow‐up imaging and clinical examinations continue to show no evidence of recurrence at 13 months following lung resection. She will continue to be monitored with regular follow‐up visits and radiological surveillance every 3–6 months.

## 3. Discussion

EHE is a rare malignant vascular neoplasm with a primary disease site in the lungs representing an exceedingly uncommon case. EHE was first described in 1975 as an aggressive form of bronchoalveolar cell carcinoma. In their case report, Dail et al. [9] discussed a tumor that invaded adjacent blood vessels and small airways. Later in 1982, Weiss and Enzinger [10] coined the term EHE to describe these vascular tumors that showed features between a hemangioma and angiosarcoma. The most recent edition of the World Health Organization classified EHE as a malignant vascular tumor composed of epithelioid endothelial cells [11].

Our patient had developed multiple new pulmonary nodules 8 years after surgical resection of RCC. Initially, the presence of multiple new lung nodules contextualized in the patient′s history sparked concern for late metastatic recurrence to the lungs. Although the recurrence rate of RCC in the lungs is over 90% within the first 5 years of nephrectomy, distant recurrence becomes highly uncommon by 10 years, with only a handful of cases documented [12–16]. Nonetheless, metastatic recurrence to the lungs was not completely ruled out until EHE diagnoses were confirmed after resection, and final tissue analysis and immunophenotyping of the lesions were performed by pathology.

Given the capped clinical familiarity of pulmonary EHE, misdiagnosis is common and prognostic estimations are vague. Consistent with other vascular tumors, pulmonary EHE typically manifests multifocally, and radiographic findings appear with three different subtypes: multiple pulmonary nodules, as seen with our patient; multiple pulmonary reticulonodular opacities; or diffuse infiltrative pleural thickening [5–7]. These three patterns of thoracic EHE imaging mimic metastatic behavior, and the question of primary versus metastatic carcinomas is often raised. Other differential diagnoses are malignant mesothelioma, epitheliod sarcoma, and pseudomyogenic hemangioendothelioma [17]. In these cases, a biopsy and immunohistochemistry to see consistent expression of endothelial differentiation markers are required for definitive diagnosis [18]. The presence of certain markers, such as mitotic count and growth patterns, can act as diagnostic clues.

As EHE is quite rare, the etiology of pulmonary EHE is still up for debate. Gordillo et al. [19] has reported that monocyte chemo‐attractant Protein‐1 is essential for EHE proliferation and may play a role in stimulating angiogenic behavior from endothelial cells, leading to the development of these lesions. Many clonal abnormalities in tumor cells have also been investigated. According to Boudousquie et al. [20], unbalanced translocations between chromosomes 7 and 22 and the loss of chromosome Y are found in tumor cells and are of interest. Errani et al. [21] has focused on genes known for their role in oncogenesis that may be involved, such as CAMTA1 on chromosome 1p36.23 and WWTR1 on chromosome 3q25. Their findings were that WWTR1‐CAMTA1, a gene fusion product, is typically found in EHE tumor cells. Further studies are required for a better understanding of the development of EHE.

Diagnosis of EHE relied on immunohistochemical analysis of our patient′s tumor and positive expression of CAMTA1, ERG, CD31, and CD34. These confirmed the EHE diagnosis, given that ancillary studies using CAMTA1 expression are currently the most reliable tool in diagnosing EHE [22, 23]. Despite the variability in clinical presentation and behavior, the immunohistochemical profile of EHE is highly consistent because of the WTR1‐CAMTA1 gene fusions that reside in 90% of EHE [17]. In addition to the expression of CAMTA1, the vascular marker ERG and the endothelial differentiation markers CD31 and CD34 are effective diagnostic resources used to confirm EHE specifically [17, 22, 24].

Our patient was asymptomatic at the time of presentation. Some have reported worse prognosis observed with respiratory symptoms or systemic systems such as fever, weight loss, or fatigue [25, 26]. Generally, the extent of malignant disease is deduced from both the presence of any systemic symptoms and from preoperative imaging delineating tumor size, invasion into adjacent structures, or any evidence of metastasis [3, 23, 27]. However, although both the clinical manifestation and its potential for malignancy can vary, pulmonary EHE development is often accompanied by a lack of symptoms [17]. When EHE is localized and complete resection is possible, 5‐year survival rates are reported to be over 90% [28]. However, once the tumor invades bilaterally or into the pleura, life expectancy was found to be significantly reduced [27, 29, 30]. Pulmonary EHE has exhibited an increased disposition for systemic involvement, and by the time of diagnosis, a large portion of pulmonary EHE cases present metastatically [17]. Further convoluting clinical assessment is the lack of validated prognostic factors to help aid treatment approach. Although some studies suggest that pleural hemorrhagic effusions, hemoptysis, cough, male sex, weight loss, and anemia are poor prognostic factors, these were only studied retrospectively and lacked unanimous validation [3, 28]. This highlights the importance of further data collection and documentation of such incidents to improve outcomes for patients.

There has been a lack of consensus on targeted treatment and surveillance, making standard guidelines for localized tumors the preferred approach. However, global attempts to establish an EHE protocol were documented during a meeting of the European Society of Clinical Oncology [17]. Complete surgical resection with negative oncological margins (R0) was recommended for localized EHE. Resections with R1 margins may be pursued if done in conjunction with postoperative radiation therapy.

Although complete surgical resection is proposed as the treatment of choice for localized EHE, a gap persists in the care of nonsurgical patients. If patients are poor surgical candidates because of severe comorbidities, or complete R0/RI resections are not anatomically or technically feasible, recommendations range widely. These nonsurgical options include surveillance, systemic treatment, or definitive radiation therapy. A previous study detailed a patient with aggressive pulmonary EHE and widespread metastases, who was treated with a systemic kinase inhibitor and anthracycline [31]. However, treatment demonstrated minimal response to disease burden. Additionally, radiation therapy for multifocal thoracic EHE has been considered palliative and dosing guidelines are not standardized [1].

Our patient was referred to a medical oncologist for recommendations on further treatment and surveillance of this rare disease. The patient will continue to follow up and will undergo interval CT scans every 3–6 months or sooner, depending on symptoms and imaging.

## 4. Conclusion

In a patient with a remote history of RCC, known to have a hematogenic metastasis pattern, diagnostic and therapeutic resection is a reasonable course. This allowed for early detection of the EHE before becoming unresectable or before the disease had metastasized. Our patient was able to achieve negative margins on pathology; however, many patients diagnosed with EHE are unable to undergo surgical resection. The scarcity of the disease, the lack of standardization of treatment, and the lack of guidance of surveillance highlight the importance of reporting individualized cases to further expand our knowledge and gain insights to further develop treatment and surveillance strategies.

**Nomenclature**
EHE: epithelioid hemangioendothelioma
VATS: video‐assisted thoracoscopic surgery
MLND: mediastinal lymph node dissection
RCC: renal cell carcinoma

## Author Contributions

Writing: R.P., C.O., C.S., H.L., I.S., and P.C.L. Pathological imaging: A.B.

## Funding

No funding was received for this manuscript.

## Ethics Statement

Ethical review and approval were waived for this study due to the Northwell Health Feinstein Institute′s Human Research Protection Program Guidance Document for Case Reports.

## Consent

Written informed consent has been obtained from the patient to publish this paper.

## Conflicts of Interest

The authors declare no conflicts of interest.

## Data Availability

Research data are not shared.
